# Approach to management of hypo and hyperthyroidism in Bangladesh: a nationwide physicians’ perspective survey

**DOI:** 10.3389/fendo.2023.1322335

**Published:** 2024-01-08

**Authors:** Shahjada Selim, Marufa Mustari, Tawshique Ahmed Khan, ABM Kamrul-Hasan

**Affiliations:** ^1^ Department of Endocrinology, Bangabandhu Sheikh Mujib Medical University, Dhaka, Bangladesh; ^2^ Department of Diabetic Foot Care, Bangladesh Diabetic Wound and Foot Care Hospital, Dhaka, Bangladesh; ^3^ Department of Endocrinology, Mymensingh Medical College, Mymensingh, Bangladesh

**Keywords:** thyroid disorders, hypothyroidism, hyperthyroidism, thyroid guidelines, Bangladesh

## Abstract

**Introduction:**

Thyroid disorders are common clinical conditions globally. The objective of the present study was to evaluate the physicians’ approach to the diagnosis and treatment of thyroid disorders in Bangladesh.

**Methods:**

The present nationally representative cross-sectional study was conducted among 662 physicians of different levels (general practitioners as well as specialists) from different hospitals in Bangladesh from January to June 2023. A self-administered semi-structured questionnaire including information about demographics and practice patterns for the diagnosis and management of thyroid disorders was used for data collection. Descriptive statistics were used to analyze the data.

**Results:**

The American Thyroid Association (ATA) guidelines were the most commonly followed guidelines for the diagnosis and management of thyroid disorders (60%), followed by the American Association of Clinical Endocrinologists (AACE) guidelines (18%) and the European Thyroid Association (ETA) guidelines (9%). Serum TSH, free T3 and free T4 levels were the most frequently used diagnostic tests for the evaluation and follow-ups of both hypothyroid and hyperthyroid states in adults, pregnant women and children, followed by total T3 and total T4 levels. Other tests, such as anti-TPO antibody, anti-TG antibody, anti-TPO, ultrasound scan of the thyroid gland, etc., were rarely used by the participating physicians. Levothyroxine at a dose of 25 to 50 mcg and carbimazole at a dose of 30 to 45 mg were the most frequently used drugs for hypothyroid and hyperthyroid patients, respectively. Almost 65% of the physicians suggested routine thyroid function tests before surgery. In addition, more than 90% of the physicians agreed that thyroid screening for pregnancy, neonates, school children and adults would be mandatory.

**Conclusion:**

The majority of the physicians participating in our study followed relevant guidelines for the diagnosis and management of thyroid disorders in Bangladesh. However, there are still some gaps to be improved, as a good number of physicians did not follow specific guidelines for these disorders.

## Introduction

Thyroid disorders are among the most prevalent clinical conditions worldwide. These include a wide range of clinical conditions ranging from asymptomatic subclinical hypothyroidism to overt hyperthyroidism.

Among these disorders, hypothyroidism, whether subclinical or overt, is the most common, especially among women. Hypothyroidism initially manifests as an increase in serum TSH concentration along with normal serum free T4 and tri-iodothyronine (T3) concentrations (subclinical hypothyroidism), which is followed by a decrease in serum free T4 concentration. Subclinical hypothyroidism usually remains asymptomatic, while overt hypothyroidism presents with a variety of nonspecific symptoms and clinical signs resulting in significant morbidity. The major cases of hypothyroidism are either dietary iodine deficiency or chronic autoimmune diseases, including atrophic autoimmune thyroiditis or goitrous autoimmune thyroiditis (Hashimoto’s thyroiditis). Epidemiological studies suggest that the global prevalence of spontaneous hypothyroidism ranges between 1 and 2%, with a female predominance (1, 2). However, there is no nationally representative population-based data regarding the prevalence of thyroid disorders among individuals of Bangladesh. A recent facility-based study including a few occupations group reported that almost 7% of the individuals had hypothyroidism (3). Similarly, in India, a neighboring south-east Asian country reported a high prevalence of thyroid disorders, almost 20% and hypothyroidism being the most common form of thyroid disorder (4). Regarding the burden of goiter and iodine deficiency, Bangladesh has experienced a significant decline in last few decades. The prevalence of goiter reduced from 55% in 1993 to almost 7% in 2005. However, the prevalence of iodine deficiency remained high (almost 40% among school-age children and 42% nonpregnant, nonlactating women) (5).

On the other hand, the major biochemical abnormality in hyperthyroidism is initially a suppressed TSH concentration along with a normal serum total T4 concentration (subclinical hyperthyroidism), which is followed by an increased serum total T4 concentration (overt hyperthyroidism). The most common causes of hyperthyroidism include Graves’ disease, toxic multinodular goitre, toxic thyroid adenoma, thyroiditis (viral or autoimmune) and drugs, including iodine and amiodarone. Different studies suggest that almost 0.5 to 2% of the global population suffers from hyperthyroidism, and the prevalence is ten times higher in women than in men (1, 6).

Several guidelines have been established by different clinical organizations for the management of thyroid disorders in different countries, including the American Thyroid Association (ATA), American Association of Clinical Endocrinology (AACE) and European Thyroid Association (ETA) (7–11). Globally, thyroid disorders are most commonly managed in primary care settings. Studies have shown that discrepancies are often seen in the clinical management of thyroid disorders in real-world settings, especially in developing countries. For example, two studies investigating clinical practice patterns in the management of primary hypothyroidism (12) and Graves’ disease (13) demonstrated both alignment and focal divergence from the available clinical practice guidelines. Despite having a high burden of patients suffering from thyroid disorders in Bangladesh (3, 14, 15), there is hardly any study investigating the practice pattern of physicians for management of these disorders. Moreover, following the appropriate guideline for all the time in a resource poor setting like Bangladesh is not always feasible. As majority of the patients do not have insurance coverage and a vast majority of the treatment expenditure comes from out-of-pocket of the patients, they often cannot bear the high expenditure of the thyroid investigation and treatment. This often results in breach of the standard guideline for management. Hence, the objective of the present study was to investigate the practice pattern of management of thyroid disorders among physicians in Bangladesh.

## Materials and methods

### Study design and participants

The present study was a cross-sectional study conducted in both government and private hospitals in Bangladesh from January to June 2023. All registered physicians who were directly involved in patient care were considered the study population.

The sample size for the present study was calculated from the following formula: n = z^2^p(1-p)/d^2^, where z = 1.96 for the 95% confidence level, p = estimated prevalence, which was considered to be 0.5 for the present study, and d = precision of error, which was considered to be 0.05 for the present study. As a multicenter study, we considered a design effect of 1.5 and a response rate of 90%. The formula provided that a total of 640 participants would be sufficient for the present study.

A snowball sampling method was used for the inclusion of the participants in the present study. The inclusion criterion was registered physicians in Bangladesh who were directly involved in patient care. Initially, the physicians who were within the familiar network of the investigators were approached for participation. After that, the participants were requested to disseminate the questionnaire within their familiar network. Thus, a final number of 662 physicians were recruited.

### Data collection

A semi-structured pretested questionnaire was used for data collection. The questionnaire had two parts containing sociodemographic information about the participants and information about their practice regarding the management of thyroid disorders. The questionnaire was prepared based on the existing guidelines and previous literature (7–13). Questions included the preferred guidelines for diagnosing and managing thyroid disorders, laboratory tests ordered for the diagnosis of these disorders in nonpregnant adults, pregnant women and children, and management options for these orders. Finally, they were asked for their perception regarding universal thyroid testing for couples, neonates, children and the adult population. The questionnaire was created in Jotform and the link was emailed to the selected physicians. A total of 976 physicians were approached and among them 662 responded (response rate 67.8%).

### Statistical analysis

All statistical analyses were carried out using Stata version 17.0. Summary statistics including frequency with percentage for categorical variables and mean with standard deviation (SD) for continuous variables were used for responses to each question.

## Results

### Sociodemographic characteristics

A total of 662 registered physicians participated in the present study. Among them, almost 28.5% were from the capital city of Dhaka, and the rest were from all over the country. The majority of the participants practiced internal medicine or its specialties including endocrinology, cardiology, nephrology, pulmonology etc. (66.6%) while others practiced surgery and its specialties. The clinical experience of more than half of the participants was less than ten years. Almost 58% of the participating physicians encountered less than 20 patients a day, and a similar number of physicians encountered less than five patients a day with thyroid disorders (**Table 1**).

**Table 1:** Sociodemographic characteristics of the participants (n = 662).

Characteristics	n	%
Location of practice		
Inside Dhaka city	187	28.46
Outside Dhaka city	470	71.54
Specialty		
Medicine and allied	441	66.62
Surgery and allied	221	33.38
Subject of the practitioners		
Endocrinology	154	23.26
Internal Medicine	78	11.78
Gynecology & Obstetrics	221	33.38
Nuclear Medicine	28	4.23
Cardiology	20	3.02
Nephrology	9	1.36
Psychiatry	3	0.45
ENT	34	5.14
Head, Neck Surgery	78	11.78
Otolaryngology	37	5.59
Experience of clinical practice		
<5 years	126	19.03
5-10 years	250	37.76
10-15 years	158	23.87
>15 years	128	19.34
Daily number of patients		
<20	390	58.91
>20	203	30.66
Daily number of patients with thyroid problem		
<5	388	58.61
>5	130	19.64

### Preferred guidelines for thyroid disorders

**Figure 1:**
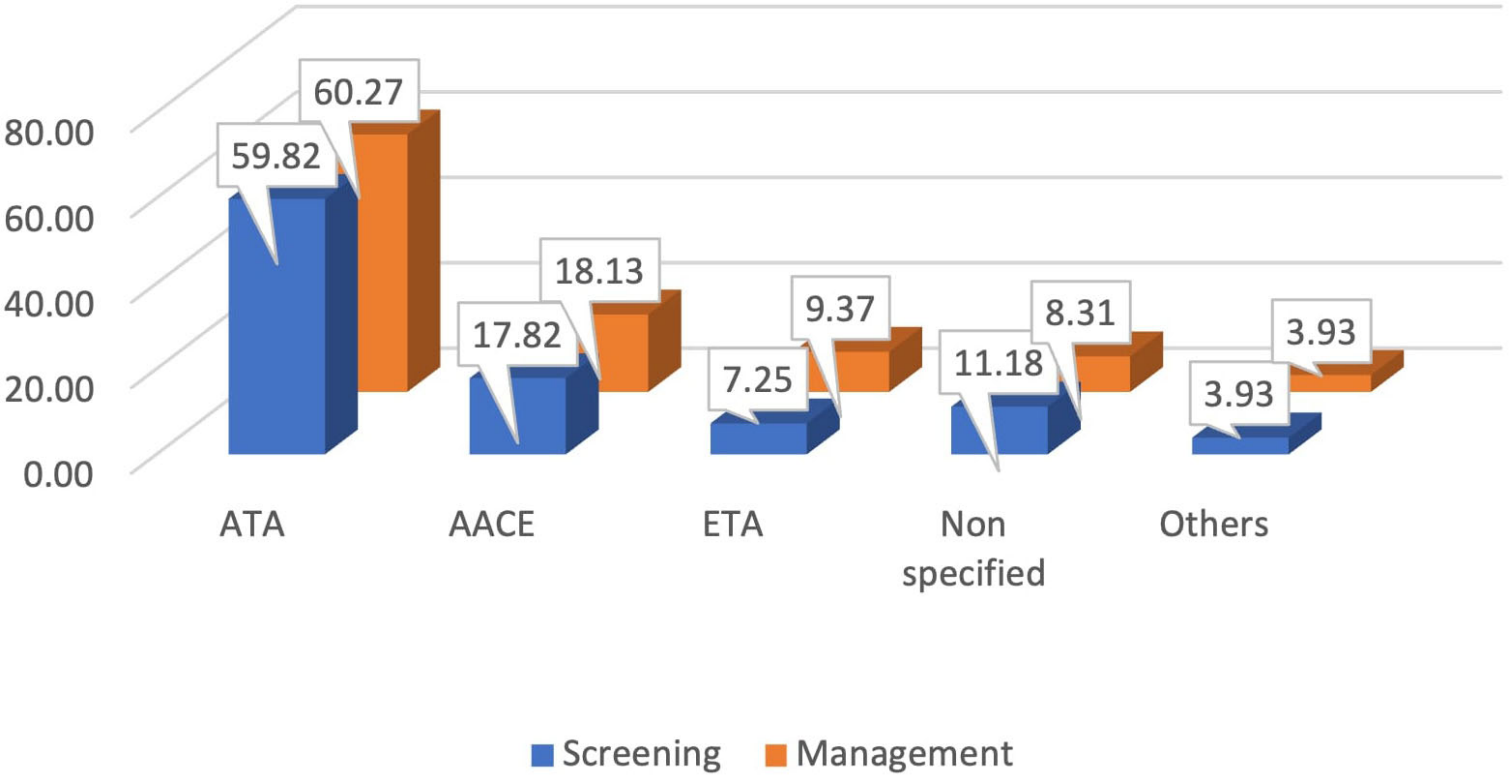
Preferred guidelines by physicians for diagnosis and management of thyroid disorders.

Almost 60% of the participants reported that they followed the American Thyroid Association (ATA) guidelines for screening as well as for managing patients with thyroid disorders in regular practice. The American Association of Clinical Endocrinology (AACE) guidelines and the European Thyroid Association (ETA) guidelines were followed by almost 18% and 9% of the physicians, respectively. Almost 11% of the participants reported that they did not follow any specific guidelines for screening, while 8.3% reported that they did not follow any guidelines for the management of thyroid disorders (**Figure 1**).

### Diagnosis of thyroid disorders

**Figure 2:**
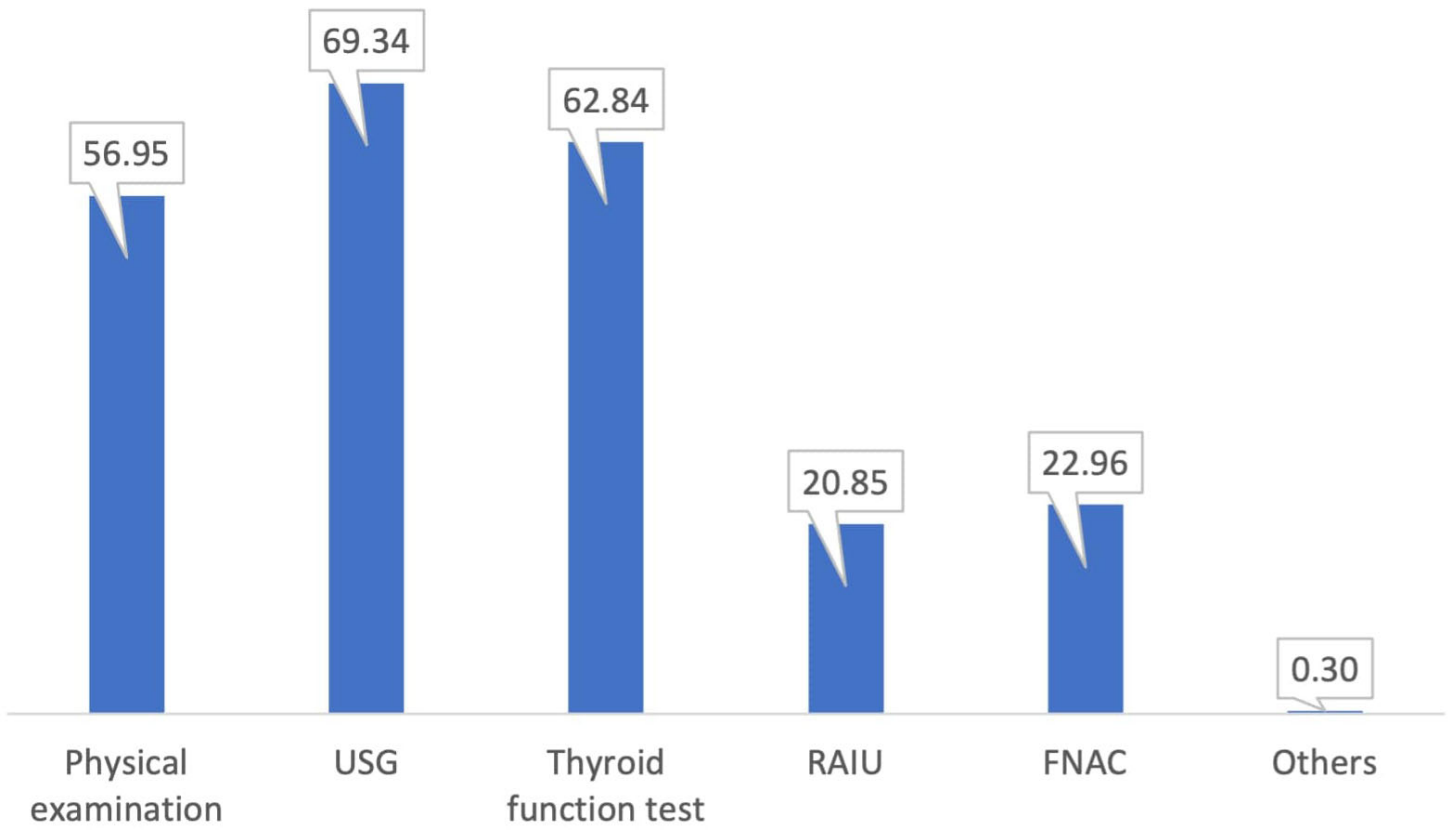
Preferred evaluation process of the physicians for a patient with an enlarged thyroid gland.

Almost 56% of the physicians participating in the present study stated that they would perform physical examination for evaluation of the patients presenting with an enlarged thyroid gland, almost 70% would perform an ultrasound scan of the gland, and 63% would perform thyroid function test. In addition, almost 21% of the physicians requested a radioactive iodine uptake assay, and 23% requested an FNAC of the enlarged gland (**Figure 2**).

**Figure 3:**
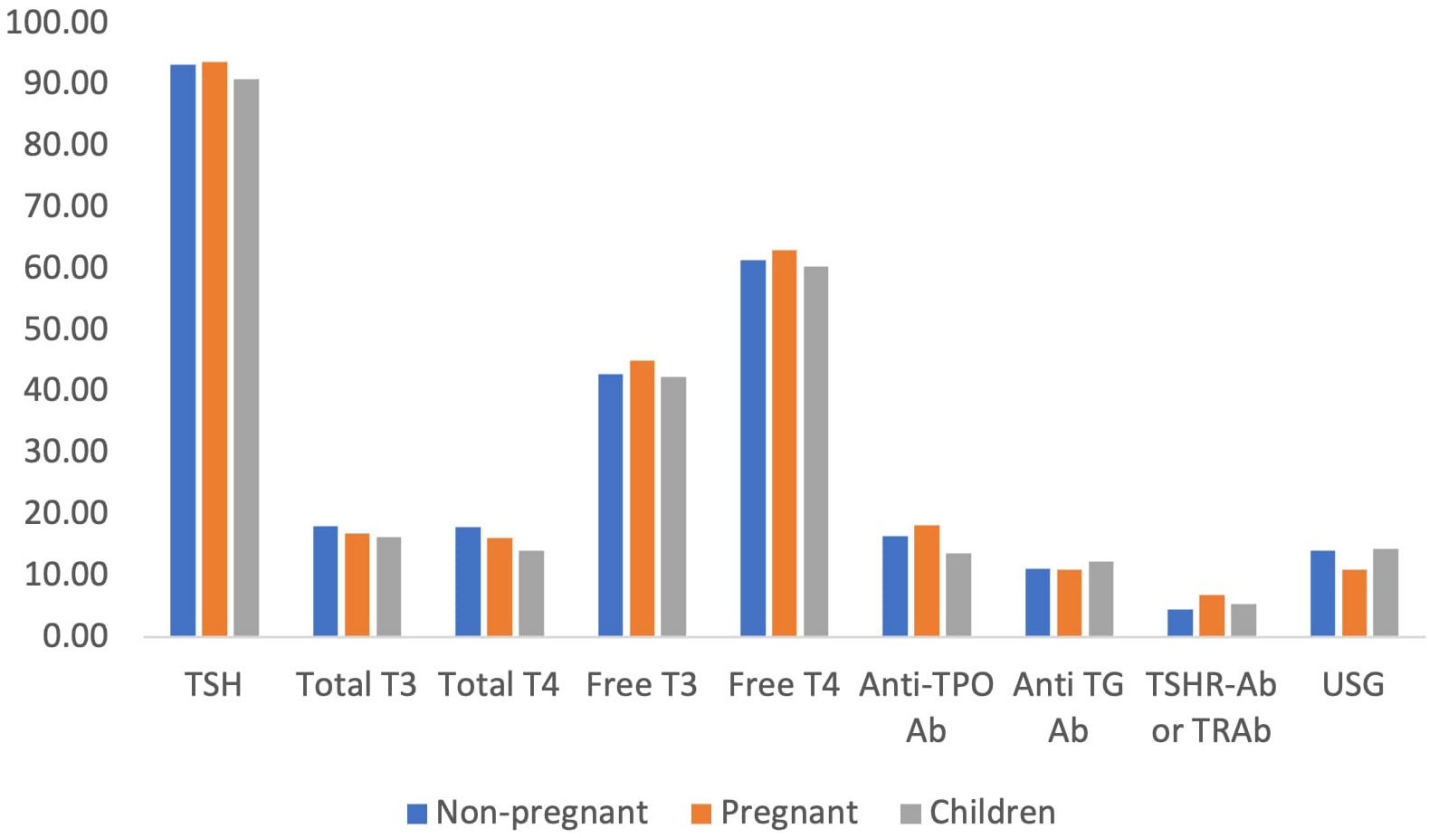
Preferred laboratory tests for diagnosis of hypothyroidism.

The serum level of thyroid stimulating hormone (TSH) was the most frequently reported test for the diagnosis of hypothyroidism in both adults and children by physicians. More than 90% of the physicians reported that they would request the serum TSH level in suspected cases of hypothyroidism, followed by serum free T4 (60%) and free T3 (43%). Other tests, including anti-thyroid antibody, anti-TSH receptor antibody and ultrasound of the thyroid gland, were not very commonly requested tests for the diagnosis of hypothyroidism (**Figure 3**).

**Figure 4:**
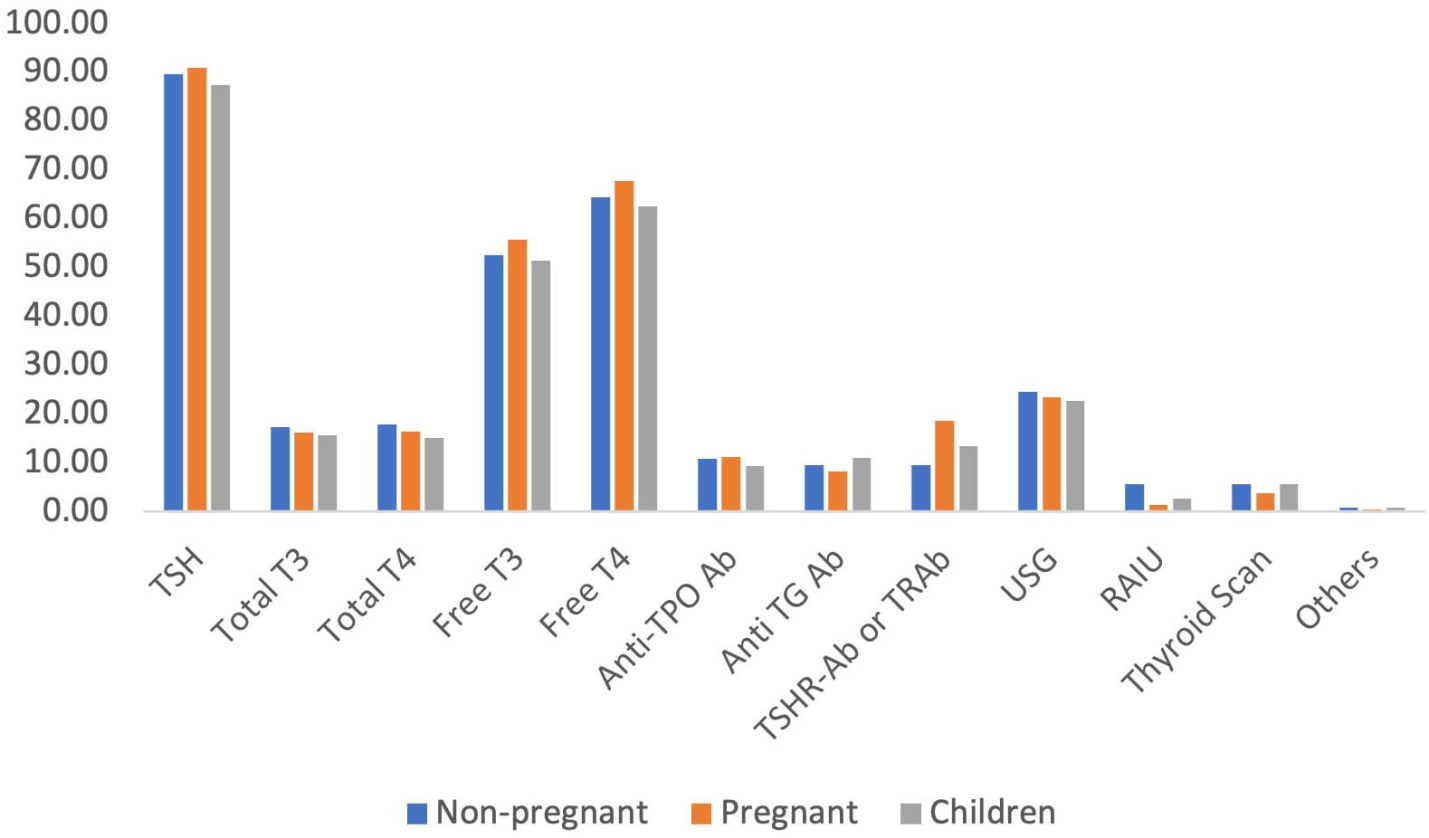
Preferred laboratory tests for the diagnosis of hyperthyroidism.

Similarly, regarding hyperthyroidism, the serum TSH level was the most commonly used diagnostic test reported by almost 90% of the participants. Other commonly reported tests were serum free T4, free T3 and ultrasound scan of the thyroid gland (**Figure 4**).

The majority of the physicians preferred The Institute of Nuclear Medicine and Allied Sciences (INMAS) or other private facilities for the diagnostic tests.

### Management of thyroid disorders

**Figure 5:**
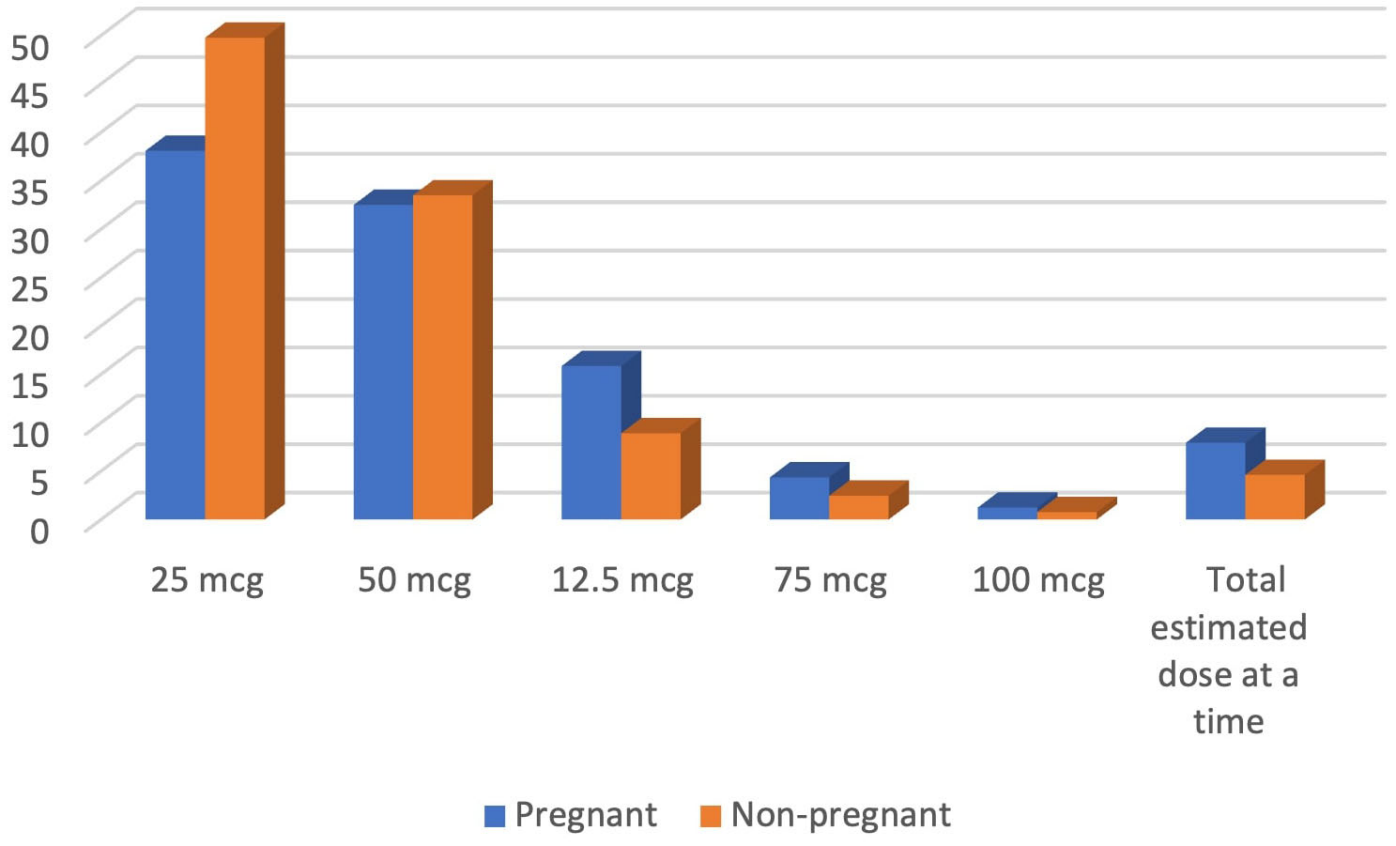
Preferred dose of levothyroxine for management of hypothyroidism.

For managing hypothyroidism in both pregnant and nonpregnant adults, the majority of the physicians reported that they would initiate levothyroxine at an initial dose of 25 mcg (50% for nonpregnant adults and 38% for pregnant women), which was followed by a dose of 50 mcg provided that their cardiovascular status was normal. Almost 4.6% of the physicians stated that they would initiate the total estimated dose at a time for nonpregnant adults, while almost 8% stated they would do the same for pregnant women with hypothyroidism (**Figure 5**).

**Figure 6:**
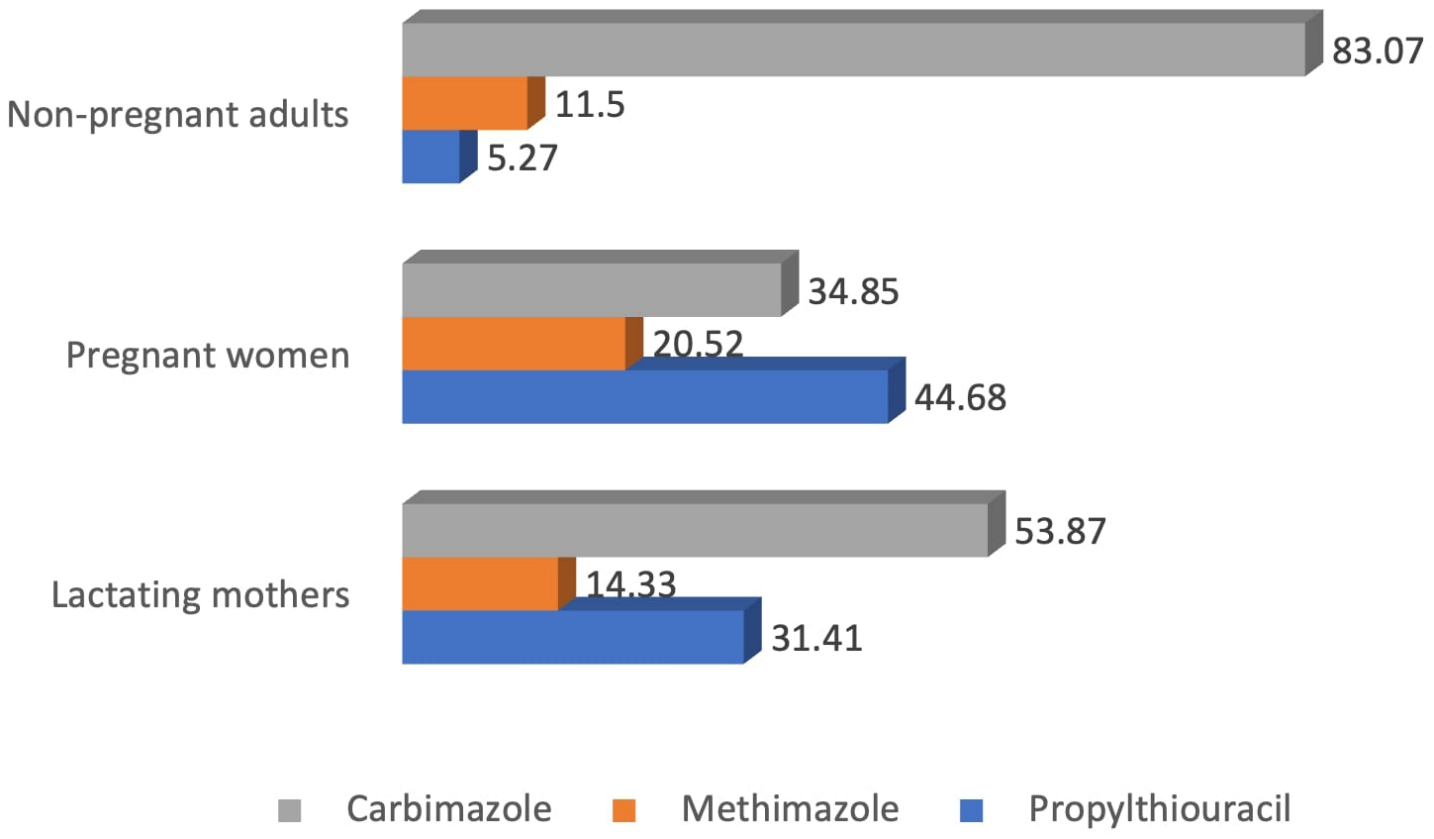
Preferred drugs for the management of hyperthyroidism.

On the other hand, carbimazole was the most common drug of choice among the physicians for the management of hyperthyroidism in nonpregnant adults (83%) and lactating mothers (54%). However, for pregnant women, the most common drug of choice was propylthiouracil (44.7%), followed by carbimazole (34.8%) (**Figure 6**).

**Figure 7:**
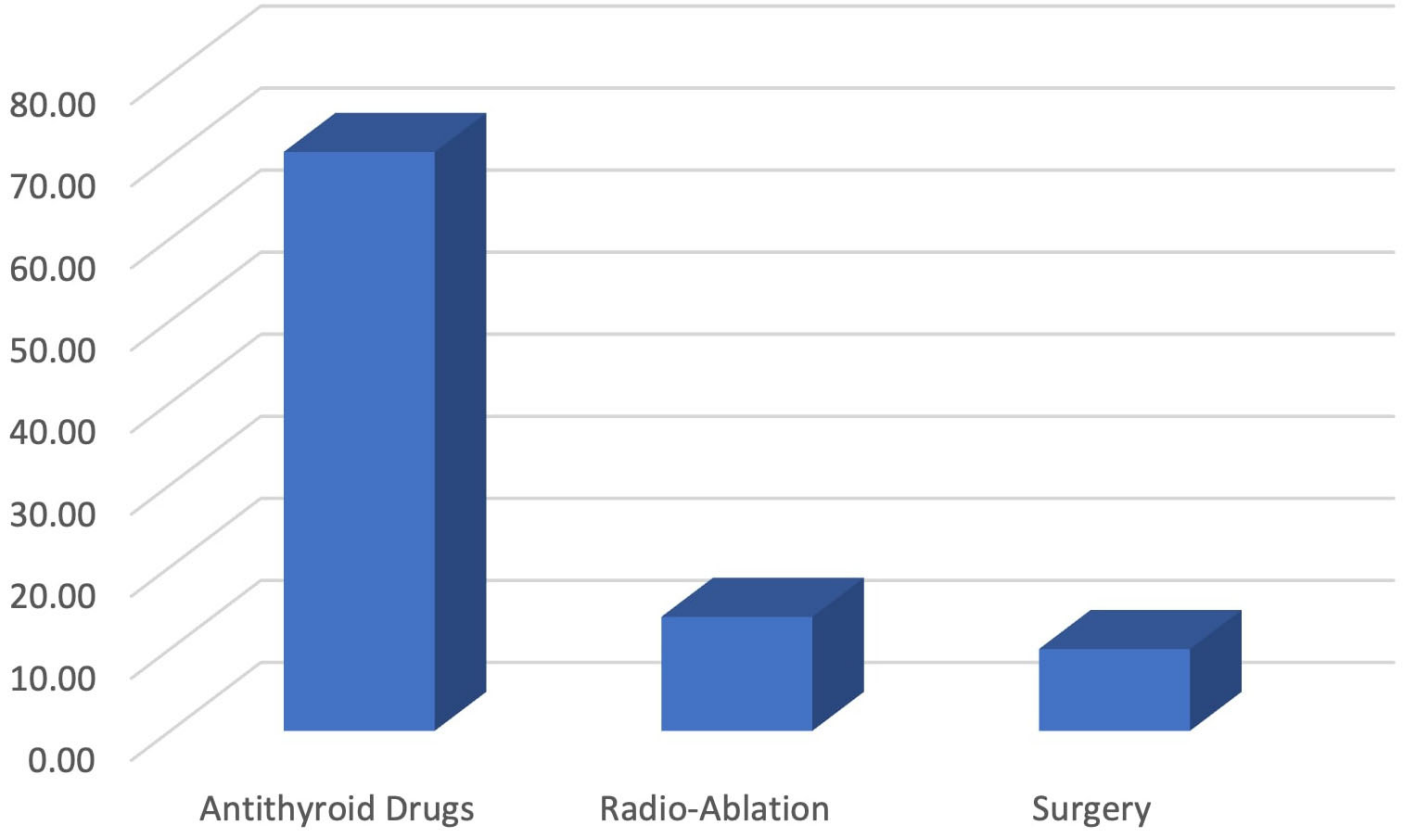
Preferred management option for Graves’ disease.

For management of Graves’ disease, almost 70% of the physicians preferred antithyroid drugs, while 14% preferred radio-ablation of the thyroid gland and 10% preferred surgical resection of the gland. In this case, surgeons preferred surgical removal of the gland to medical treatment compared to medicine specialists (14% vs 6.2%) (**Figure 7**).

### Follow-up of patients with thyroid disorders

**Figure 8:**
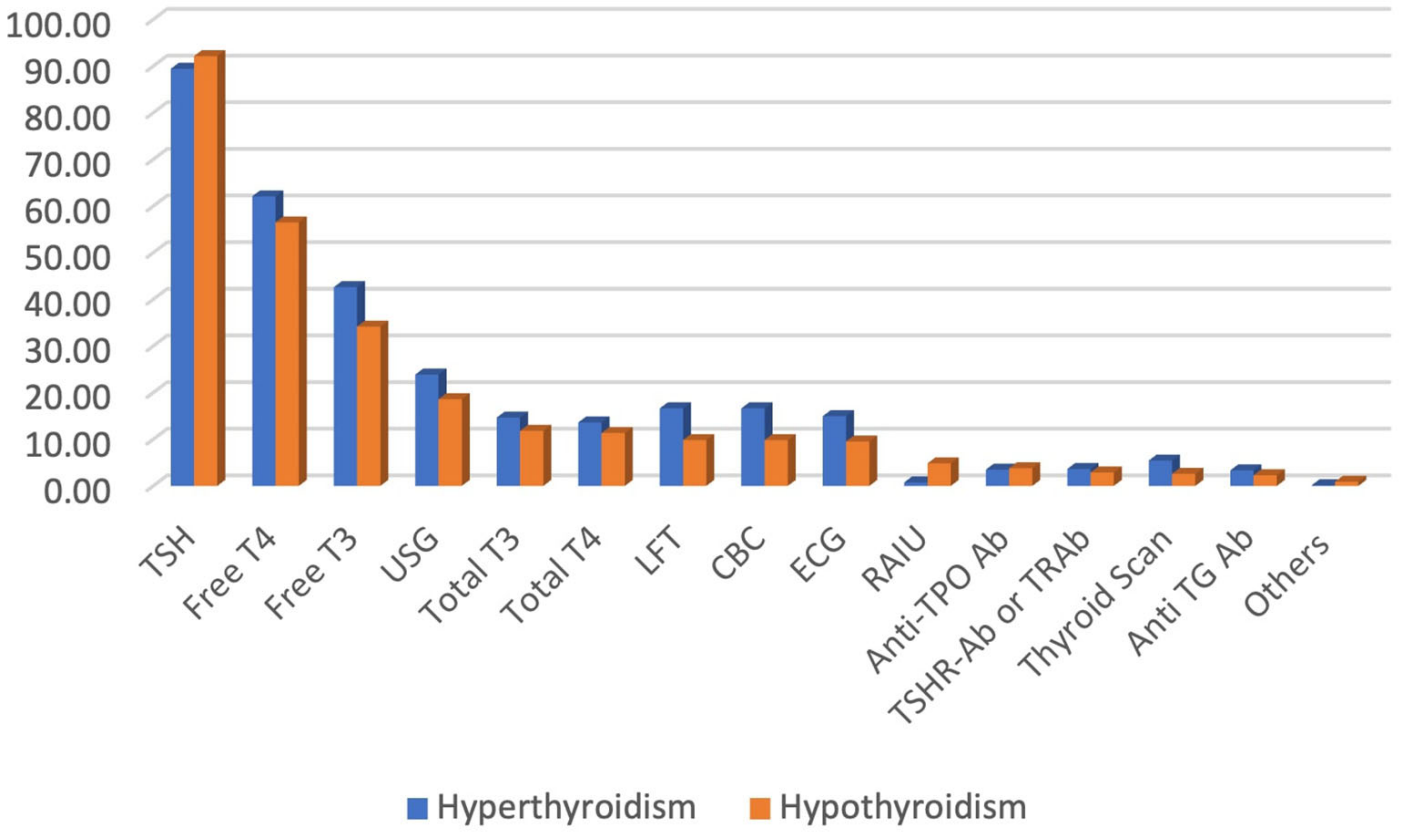
Preferred tests for follow-up of patients with thyroid disorders.

Almost two-thirds of the physicians reported that they follow-up with their patients with thyroid disorders at least once every six to twelve weeks. For follow-up of both hypo- and hyperthyroid patients, serum TSH level remained the most commonly used laboratory test among the physicians (92% and 89% for hypo- and hyperthyroidism, respectively), followed by serum free T4 and free T3 (**Figure 8**).

### Attitude toward routine thyroid tests

**Figure 9:**
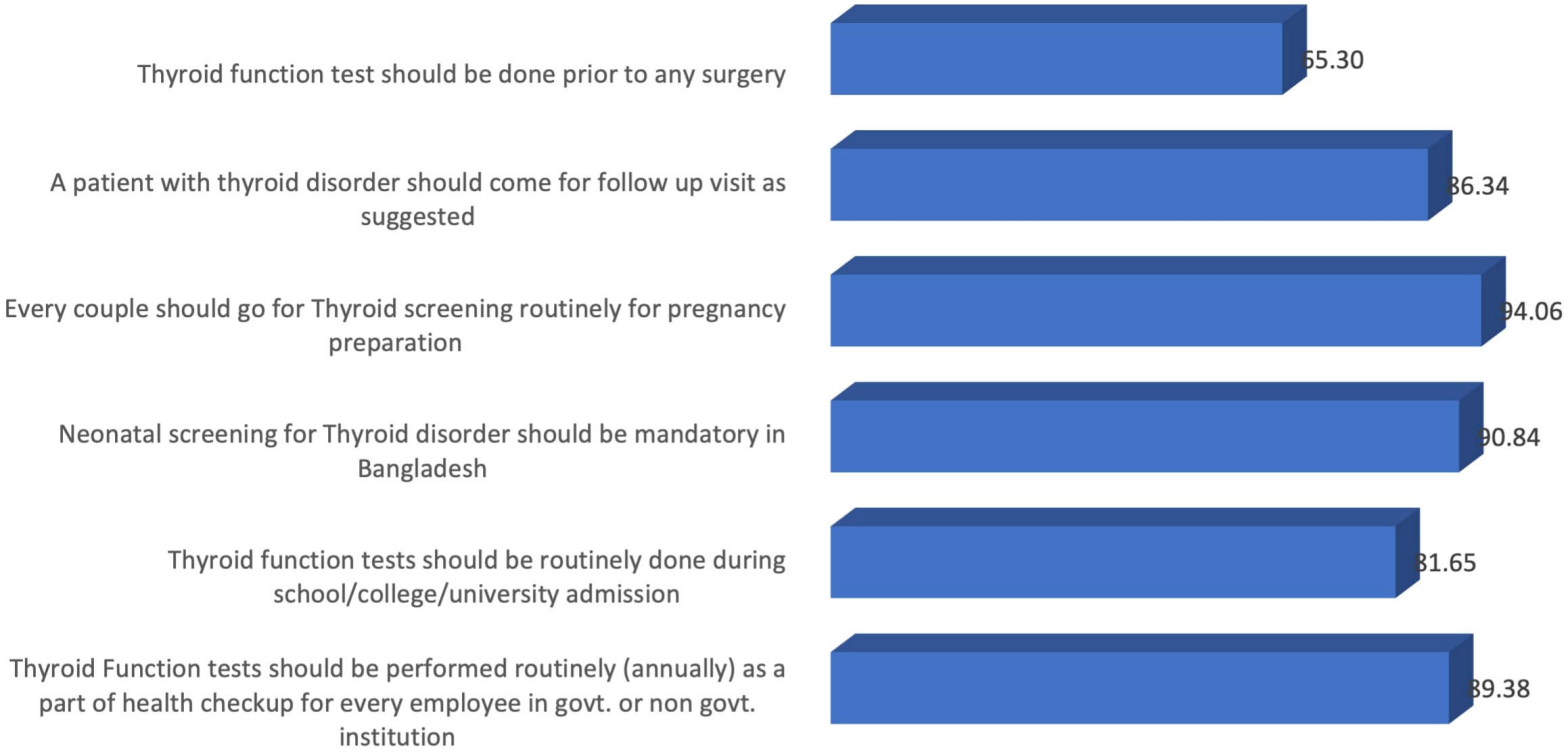
Attitudes toward routine thyroid tests among physicians.

Almost 65% of the participating physicians believed that thyroid function tests should be routinely performed before surgery. Moreover, the majority of the participants agreed that thyroid function tests should be suggested for couples, neonates, children entering educational institutions, and adults (**Figure 9**).

## Discussion

The present study provides an overview regarding the practice pattern of physicians in Bangladesh for the diagnosis and management of thyroid disorders. This study includes a demographically diverse collection of physicians from all over the country. Our study found that the majority of physicians follow at least one established guideline for the diagnosis and management of hypothyroidism or hyperthyroidism. The American Thyroid Association (ATA) guideline remains the most commonly followed guideline by the participating physicians. Other frequently followed guidelines were the American Association of Clinical Endocrinology (AACE) guidelines and the European Thyroid Association (ETA) guidelines. However, there is an issue of concern that a number of physicians reported that they did not follow any specific guidelines, which might result in their substandard care for the patients. Some previous studies conducted in multiple countries reported a similar finding. For example, a study by Burch et al. reported that almost 90% of physicians practice concordant with the guidelines for hypothyroidism (12). A similar finding was presented in a study regarding the management of Graves’ disease (13).

In the current world of rapid advancement in medical science, clinical practice guidelines can provide updated knowledge regarding optimum management of diseases. Guidelines from the responsible authorities of the respective subjects can accelerate the recent trend of practicing evidence based medicine. A study regarding the role of thyroid disease guidelines in provider education and in subsequent patient care decisions concluded that exposure to the relevant guidelines resulted in better patient care and clinical outcome (16).

In our study, the majority of the physicians reported that they would suggest serum TSH, free T4 and free T3 levels for the diagnosis of both hypo- and hyperthyroidism. However, it is not recommended to use free T3 levels for the diagnosis of hypothyroidism in the ATA guidelines. In addition, it was recommended that in patients with subclinical hypothyroidism, TPO Ab should be tested (12), which was mostly ignored by our participants. A study from the USA reported that almost one-third of physicians would suggest thyroid scans in patients with Graves’ disease (13), which was very negligible in our study, ranging from 2-5%. In addition, our participants mostly used serum TSH levels for the follow-up of patients with thyroid disorders. Though TSH alone can be used for follow-up of hypothyroidism, it is recommended that both free T4 and TSH levels should be evaluated for monitoring the patients with hyperthyroidism (12, 13), which was followed by almost half of the participants.

According to the guidelines, patients should be treated if the serum TSH level is above 10 mIU/L, and levothyroxine alone would be enough for the treatment. Moreover, age-specific and pregnancy-specific target TSH levels should be used for the management of these patients, and treatment should be reviewed and adjusted after four to eight weeks of initiation (7). In our study, it was found that the majority of the physicians used levothyroxine at a recommended dose for the management of hypothyroidism. On the other hand, carbimazole was the most common drug of choice among the physicians for the management of hyperthyroidism in nonpregnant adults, while propylthiouracil was the most common drug for pregnant women. Despite the fact, a significant percentage of your respondents would still use carbimazole or methimazole in pregnancy, which goes against ATA guidelines (7). Adequate awareness and training of the physicians is required in this regard to refrain themselves from these practices. In addition, the majority of the physicians preferred antithyroid drugs for the treatment of Graves’ disease, while others preferred radioablation of the thyroid gland or surgical resection of the gland. In our study, almost 10% of the respondents preferred surgery for management of Graves’ disease. This proportion is higher compared to the physicians from Europe and America (12, 13). The underlying cause of this popularity of surgery might derive from the fact that we included a handful number of physicians from surgical specialty in our study who preferred surgical management to medical treatment for Grave’s disease.

In our study, we found that the majority of the physicians had a positive attitude toward universal thyroid screening for couples planning for pregnancy, neonates, children and adults. As thyroid disorders impose an enormous burden of morbidity on the population, it might be helpful for early diagnosis and treatment of these disorders and reducing the long-term complications.

Following clinical guidelines has a positive influence on patient care. However, a handful number of physicians in Bangladesh are reluctant to follow the guidelines adequately for management of thyroid disorders, as our study found. In this context, easy accessibility and adequate training on the practical applicability of the available guidelines may improve the situation. Similar suggestion would also be applicable to other developing countries where healthcare system is vulnerable.

As one of the very few studies, ours provides a bird’s-eye view of the practice pattern of physicians in Bangladesh regarding the diagnosis and management of thyroid disorders. However, the study has several limitations. First, the study adopted a subjective evaluation using a self-administered questionnaire, where a scope of social desirability bias always remains. Moreover, the findings might not be generalizable for all physicians of the country, as representativeness from different specialties was not ensured.

## Conclusion

In summary, our survey among the physicians of Bangladesh regarding the management of thyroid disorders depicts the current practice patterns and demonstrates both alignment and focal deviation from the guidelines. Easy accessibility and a multidisciplinary approach for training on the relevant guidelines on thyroid diseases is suggested for standardization and optimization of patient care.

## Data availability statement

The raw data supporting the conclusions of this article will be made available by the authors, without undue reservation.

## Ethics statement

The studies involving humans were approved by Bangabandhu Sheikh Mujib Medical University. The studies were conducted in accordance with the local legislation and institutional requirements. The participants provided their written informed consent to participate in this study.

## Author contributions

SS: Conceptualization, Investigation, Methodology, Project administration, Supervision, Writing – original draft, Writing – review & editing. MM: Conceptualization, Data curation, Formal analysis, Investigation, Methodology, Resources, Writing – review & editing. TK: Data curation, Investigation, Methodology, Writing – review & editing. AH: Conceptualization, Data curation, Investigation, Writing – review & editing, Methodology.
